# Correlation of CT-derived pectoralis muscle status and COVID-19 induced lung injury in elderly patients

**DOI:** 10.1186/s12880-022-00872-9

**Published:** 2022-08-12

**Authors:** Pei Ying-hao, Zhang Hai-dong, Fang Yuan, Liu Yong-kang, Liang Sen, Xu Wei-long, Yang Yu-shan, Zhu Jun-feng, Zhou Hai-qi, Jiang Hua

**Affiliations:** 1 Department of Intensive Care Unit, Jiangsu Province Hospital of Chinese Medicine, Affiliated Hospital of Nanjing University of Chinese Medicine, Nanjing, Jiangsu Province China; 2 Department of Geriatrics, Jiangsu Province Hospital of Chinese Medicine, Affiliated Hospital of Nanjing University of Chinese Medicine, Nanjing, Jiangsu Province China; 3 Department of Radiology, Jiangsu Province Hospital of Chinese Medicine, Affiliated Hospital of Nanjing University of Chinese Medicine, Nanjing, Jiangsu Province China; 4 Department of Neurology, Jiangsu Province Hospital of Chinese Medicine, Affiliated Hospital of Nanjing University of Chinese Medicine, Nanjing, Jiangsu Province China; 5 Department of Endocrinology, Jiangsu Province Hospital of Chinese Medicine, Affiliated Hospital of Nanjing University of Chinese Medicine, Nanjing, Jiangsu Province China; 6 First School of Clinical Medicine, Nanjing University of Chinese Medicine, Nanjing, Jiangsu Province China

**Keywords:** Elderly, Computed tomography, Pectoralis muscle, COVID-19, Severity

## Abstract

**Objectives:**

To explore the association between CT-derived pectoralis muscle index (PMI) and COVID-19 induced lung injury.

**Methods:**

We enrolled 116 elderly COVID-19 patients linked to the COVID-19 outbreak in Nanjing Lukou international airport. We extracted three sessions of their CT data, including one upon admission (T1), one during the first 2 weeks when lung injury peaked (T2) and one on day 14 ± 2 (T3). Lung injury was assessed by CT severity score (CTSS) and pulmonary opacity score (POS). Pneumonia evolution was evaluated by changes of CT scores at T2 from T1(Δ).

**Results:**

The maximum CT scores in low PMI patients were higher than those of normal PMI patients, including CTSS1 (7, IQR 6–10 vs. 5, IQR 3–6, *p* < 0.001), CTSS2 (8, IQR 7–11 vs. 5, IQR 4–7, *p* < 0.001) and POS (2, IQR 1–2.5 vs. 1, IQR 1–2, *p* < 0.001). Comorbidity (OR = 6.15, *p* = 0.023) and the presence of low PMI (OR = 5.43, *p* = 0.001) were predictors of lung injury aggravation with ΔCTSS1 > 4. The presence of low PMI (OR = 5.98, *p* < 0.001) was the predictor of lung injury aggravation with ΔCTSS2 > 4. Meanwhile, presence of low PMI (OR = 2.82, *p* = 0.042) and incrementally increasing D-dimer (OR = 0.088, *p* = 0.024) were predictors of lung injury aggravation with ΔPOS = 2.

**Conclusions:**

PMI can be easily assessed on chest CT images and can potentially be used as one of the markers to predict the severity of lung injury in elderly COVID-19 patients.

**Supplementary Information:**

The online version contains supplementary material available at 10.1186/s12880-022-00872-9.

## Introduction

In December 2019, a novel type of coronavirus, severe acute respiratory syndrome coronavirus 2 (SARS-CoV-2) was identified and the World Health Organization (WHO) named this infection as coronavirus disease 2019 (COVID-19) [1]. According to data from WHO, as of November 18, 2021, the global cumulative number of confirmed COVID-19 cases has risen to over 254 million, and more than 5.1 million patients have died from it [2]. The majority of COVID-19 is asymptomatic or with mild symptoms including fever, cough, shortness of breath, nausea, and fatigue. However, some patients are still likely to deteriorate and develop severe pneumonia, acute respiratory distress syndrome, and even death. As indicated by recent studies, elderly patients and those with underlying comorbidities, such as cardio-pulmonary abnormalities, diabetes mellitus and obesity, are at higher risk of hospitalization and developing severe complications [3, 4].

In addition to these established risk factors, the latest studies [5–7] suggest that muscle strength and mass should be noticed. Due to progressive loss of skeletal muscle mass and strength, sarcopenia is generally encountered with elderly subjects [8]. Lower muscle mass or sarcopenia has been suggested as an independent predictor of unfavorable outcome in major surgery [9, 10], cancer [11, 12] and chronic disease [13, 14]. Muscle health, including strength and mass, assessed upon hospital admission was proved to be the predictor of hospitalization in patients with moderate to severe COVID-19 [5]. For patients that age over 50 years, engaging in physical activity more than once a week is associated with lower odds of COVID-19 hospitalization [6]. The protective effect of physical activity on COVID-19 hospitalization is explained by muscle strength. It is also reported in another study that muscle strength is an independent risk factor for COVID-19 severity in adults 50 years of age or older [7]. Chest CT is a promising way to evaluate muscle mass. It has been reported that lower mass of paravertebral muscles on CT is independently associated with ICU admission and hospital mortality [15]. Due to pressure of gravity on supine position, there is a high bias risk for paravertebral muscle mass measure. Thus, area of pectoralis muscle is not affected by a supine position of CT test. Pectoralis on cross-sectional CT images is associated with lean muscle mass, handgrip strength and sarcopenia [16]. Area of pectoralis muscle has been reported as a predictor of prolonged hospital stay and intubation in COVID-19 patients [17], but this study did not specifically consider elderly patients.

Therefore, we intended to retrospectively investigate the potential contribution of CT-derived pectoralis muscle index (PMI) in predicting clinical outcomes and COVID-19 induced lung injury severity in 116 elderly COVID-19 patients. All enrolled patients were in a designated hospital and linked to the outbreak of COVID-19 in Nanjing Lukou international airport.

## Methods

### Study design and population

**Fig. 1:**
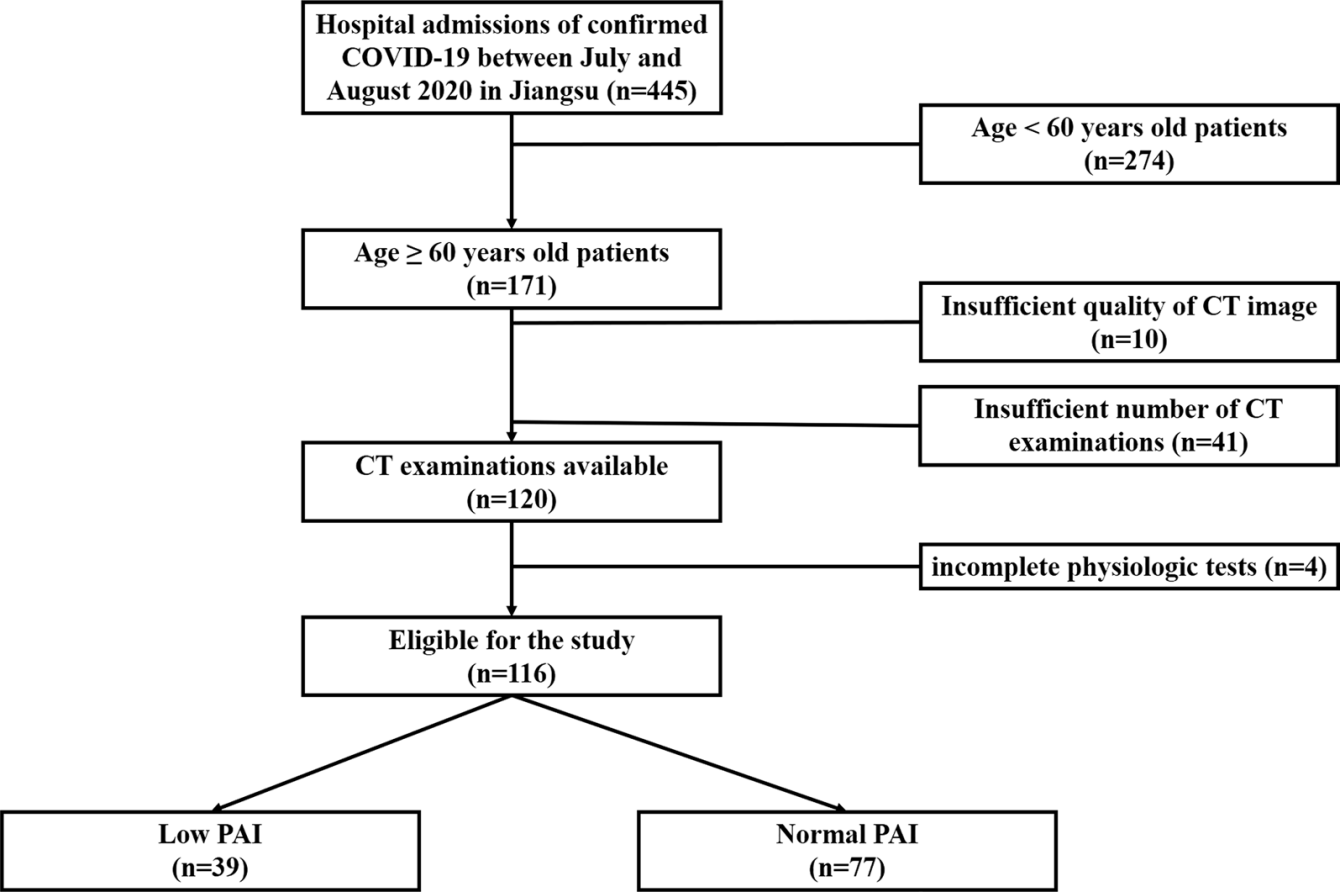
Flow diagram of the study population

Clinical Research Ethics Committee of Jiangsu Province Hospital of Chinese Medicine approved this retrospective study (2021NL-207-01) and waived written informed consent since the study was retrospective and was part of a public health outbreak investigation. All methods were carried out in accordance with relevant guidelines and regulations. We enrolled 116 elderly patients diagnosed with COVID-19 by RT-PCR test between 25 July 2021 and 15 August 2021 in Jiangsu, China. Inclusion criteria: (1) Age over 60 years, (2) confirmed by COVID-19 nucleic acid RT-PCR test, (3) linked to the recent outbreak of COVID-19 originated in Nanjing Lukou international airport, (4) underwent at least 5 times chest CT scans during the first 2 weeks of hospitalization. All enrolled patients hospitalized at Nanjing Public Health Medical Center, the only designated hospital that provides medical services for COVID-19 in Nanjing. Exclusion criteria: (1) severe illness upon admission, such as respiratory failure requiring mechanical ventilation, shock with the need for vasopressors; extracorporeal membrane oxygenation treatment, (2) incomplete clinical data, (3) insufficient CT image quality. Of the 116 elderly patients in this study, 12 lived in Nanjing, 101 lived in Yangzhou and 2 lived in Huai’an. The procedure to enroll patients was conducted as presented in Fig. 1.

### Chest computed tomography image acquisition

Chest CT images were obtained using a multi-detector CT scanner (Brilliance 64, Philips Medical Systems) at deep inspiration in the supine position. The CT scanner was dedicated only to COVID-19 patients. The CT room and CT scanner were sanitized using standard cleaning procedures. The scanning parameters were 16 × 0.75 mm slice collimation, 0.5–0.75 s rotation time, 5 mm slice thickness and 1 mm slice re-constructions, 250–300 mm field of view, 120 kV tube voltage, 50–300 mAs effective tube current–time product, and 512 × 512 matrix. All patients were under arm position in the CT scans.

### CT image analysis

**Fig. 2:**
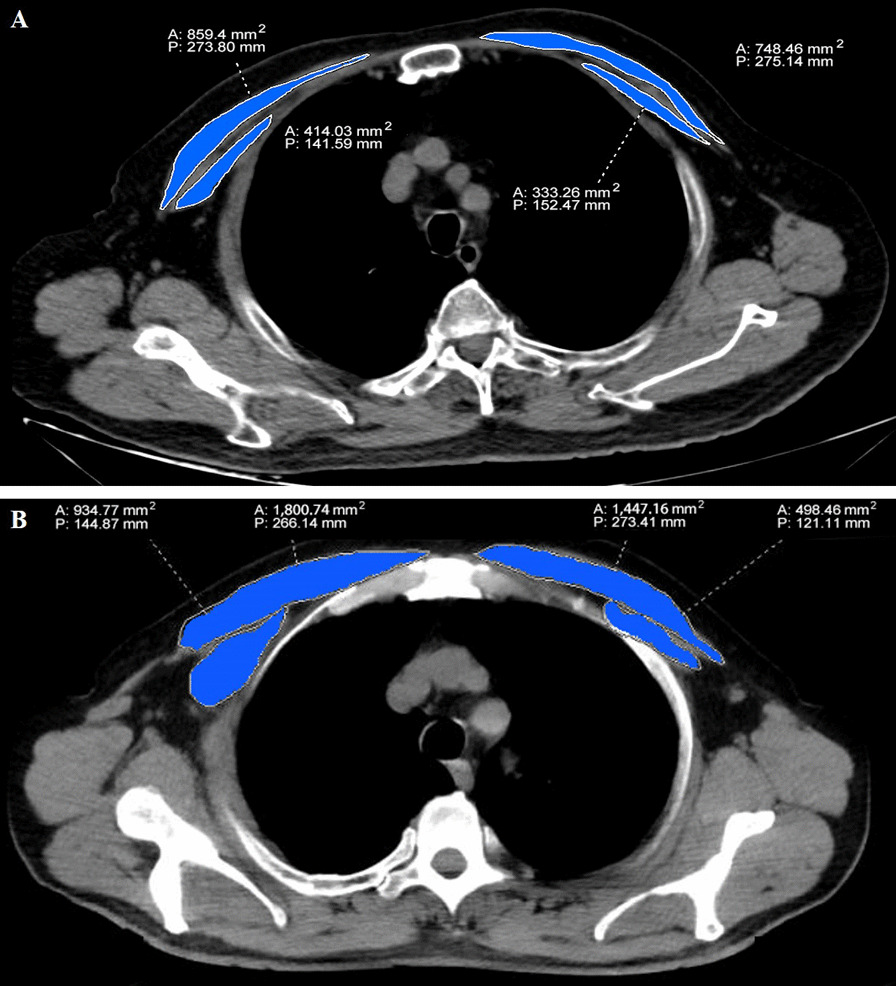
The pectoralis muscle area (PMA) measurement on chest CT image. Bilateral pectoralis muscles are colored blue (pectoralis major and minor muscles). **a** A 67-year-old male patient with COVID-19. **b** A 72-year-old male patient with COVID-19. A, Area, P, Perimeter

We extracted three sessions of their CT data, including one upon admission (T1), one during the first 2 weeks when lung injury peaked (T2) and one on day 14 ± 2 (T3). All the chest CT images were independently evaluated and measured by two physicians with over seven years of experience in critical care thoracic imaging, who were unaware of the patient’s laboratory and clinical findings. When there is disagreement, a third expert with 15-year experience adjudicated a final decision. We first measured pectoralis muscle area (PMA) in the CT image that just above the aortic arch using a free DICOM web viewer (SYNAPSE PACS,FUJI Film, China) according to previous study [17] (Fig. 2). To normalize PMA values, gender specific pectoralis muscle index (PMI) was calculated by the PMA value divided by the body surface area (BSA, m^2^). The formula of BSA in this study was according to Mosteller’s methods [18].

Then, we assessed SARS-CoV-2 induced lung injury using CT severity score (CTSS) and pulmonary opacity score (POS) [19], which have been described previously. The degree of involvement in CTSS was scored as follows: a score of 0 denoted no involvement; 1 point, < 25% involvement; 2 points, 25% to less than 50% involvement; 3 points, 50% to less than 75% involvement; and 4 points, ≥ 75% involvement. The involvement severity was evaluated separately for each lobe in CTSS 1 [20] and each six zones in CTSS 2 [21]. These six zones of CTSS 2 were the upper zone (above the carina), the middle zone (from the carina to the inferior pulmonary vein), and the lower zone (below the inferior pulmonary vein) of both right and left lung lobes. POS was scored according to the percentage of pulmonary opacity area [ground-glass opacities (GGO) or consolidation] relative to the entire lung on CT images, which presented as 0 point, ≤ 5% involvement; 1 point, 6–20% involvement; 2 points, 21–40% involvement; and 3 points, ≥ 41% involvement. Total scores of CTSS1, CTSS2 and POS ranged from 0 to 20, 0 to 24 and 0 to 3, respectively. We calculated the changes of these three scores from T2 to T1 (Δ), which reflected evolution of pneumonia. To analyze the value of the inter-observer agreement, two observers used the same method and blind to each other.

### Date collection

Demographic and clinical data were collected, including age, gender, medical history, smoking history, results from physical examinations, and laboratory results. The validity of all data was checked by two physicians (YYS and ZJF). We calculated the clinical syndrome score in each patient, which was the sum of the duration of each symptom.

### Statistical analysis

IBM SPSS Statistics (version 21.0, IBM, Armonk, NY, USA) was used for data analysis. Continuous data were presented as means and standard deviations (SDs), or medians and interquartile ranges (IQRs). Differences between groups were tested using the Kruskal–Wallis test with Mann Whitney *U* test, χ^2^ test, or Fisher exact test. A two-sided significance level of 5% was used, and 95% CIs were reported for all analyses. All patients were divided into gender-specific PMI tertiles. Low PMI was categorically defined as the smallest tertile in male and female patients respectively. Logistic regression analysis was used to estimate the relative effect of variables for levels of lung injury by calculating unadjusted odds ratios (ORs). In the univariate analysis, statistically significant variables (*p* < 0.05) were used for multivariate modeling. Interobserver agreement was evaluated by the intraclass correlation coefficient (ICC) score. The significance level was set at 0.05.

## Results

### Baseline demographic and clinical characteristics

Demographic and clinical characteristics of the 116 COVID-19 elderly patients are enumerated in Table 1. The median age was 69 years (IQR 65–74; range 60–84), and 40 (34.5%) patients were male. Fever (68.1%), cough (86.2%) and fatigue (56%) were the three most common symptoms. Hypertension (50%) and diabetes (21.6%) were the two most common underlying medical conditions. Only 17 (14.7%) patients were fully vaccinated while the numbers of unvaccinated and partially vaccinated patients were 60 (51.7%) and 39 (33.6%). The median time from illness onset to hospitalization was 4 days (IQR 2–5.75, range 1–15). The median length of hospitalization was 26 days (IQR 20.25–29, range 12–33). All patients in our study received on-demand traditional Chinese medicine treatment.

**Table 1:** Baseline characteristics of all elderly COVID-19 patients

Characteristics	All patients (N = 116)	Low PMI (n = 39)	Normal PMI (n = 77)	*p* value
Age	69(65,74)	71(66,75)	68(65,72)	0.041
Male	40(34.5)	14(35.9)	26(33.8)	0.820
BMI	24.2(22.5,26.9)	23.8(21.6,26.0)	24.9(22.7,27.1)	0.124
Current smoking	25(21.6)	15(38.5)	10(13.0)	0.002
Clinical severity score				0.543
Common	113(97.4)	37(94.9)	76(98.7)	
Severe	3(2.6)	2(5.1)	1(1.3)	
Days from T0 to T1 (days)	5(3,8)	6(3,8)	5(2,7)	0.025
Having underlying medical conditions				
Hypertension	56(48.3)	21(53.8)	35(45.5)	0.435
Diabetes	25(21.6)	8(20.5)	17(22.1)	0.846
Coronary heart disease	5(4.3)	2(5.1)	3(3.9)	1
COPD/asthma	10(8.6)	4(10.3)	6(7.8)	0.731
CKD	2(1.7)	0(0)	2(2.6)	0.550
Carcinoma history	11(9.5)	5(12.8)	6(7.8)	0.591
Vaccination status				0.485
Unvaccinated	60(51.7)	16(41)	23(29.9)	
Partially vaccinated	39(33.6)	5(12.8)	12(15.6)	
Fully vaccinated	17(14.7)	18(46.2)	42(54.5)	
Time from illness onset to hospitalization (days)	4(2,5.75)	4(2,5)	4(2,6)	0.894
Length of stay (days)	26(20.25,29)	26(21,29)	26(20,29)	0.684
Clinical syndrome score	17.5(12,28)	34(30,38)	16(11,23)	< 0.001
Fever	86(74.1)	30(76.9)	56(72.7)	0.626
Cough	102(87.9)	37(94.9)	65(84.4)	0.183
Shortness of breath	23(19.8)	7(17.9)	16(20.8)	0.718
Nausea or vomiting	28(24.1)	17(43.6)	11(14.3)	0.001
Abdominal pain or diarrhea	41(35.3)	15(38.5)	26(33.8)	0.617
Loss of smell or taste	13(11.2)	6(15.4)	7(9.1)	0.482
Stuffy nose or runny nose	28(24.1)	17(43.6)	11(14.3)	0.001
Headache or dizziness	13(11.2)	8(20.5)	5(6.5)	0.051
Fatigue	89(76.7)	34(87.2)	55(71.4)	0.058
Pharyngeal discomfort	36(31.0)	19(48.7)	17(22.1)	0.003
Medical drugs				
Traditional Chinese medicine	116(100)	39(100)	77(100)	1
Immunoglobulin	8(6.9)	3(7.7)	5(6.5)	1
Glucocorticoid	1(0.9)	0(0)	1(1.3)	1
Any antibiotics	13(11.2)	11(28.2)	2(2.6)	< 0.001
Any antivirals	41(35.3)	14(35.9)	27(35.1)	0.929
Neutralizing antibodies	6(5.2)	3(7.7)	3(3.9)	0.402
Anticoagulant	105(90.5)	37(94.9)	68(88.3)	0.330

Data are the median (interquartile range) or number of patients (percentage) unless otherwise indicated. Clinical syndrome score, the sum of each symptom duration days

The ICC value of PMA was 0.918 (95% CI, 0.884–0.943). The first tertile cut-off values of PMI in males and females were 16.4 cm^2^/m^2^ and 13.8 cm^2^/m^2^, respectively. A total of 39 patients in the first tertile of PMI value in both genders were divided into low PMI group. Low PMI patients were older than the normal PMI (*p* = 0.041). The numbers of currently smoking patients in low PMI group were higher than that in normal PMI group (*p* = 0.002). Low PMI group showed a longer day from T1 to T2 than normal group (*p* = 0.025), which indicated that low PMI patients presented a prolonged peak of lung injury. The clinical syndrome score was higher in low PMI group than that of normal group (*p* < 0.001). Nausea/vomiting, congested nose/runny nose and pharyngeal discomfort were more prevalent in the low PMI group (*p* = 0.001, 0.001 and 0.003, respectively). More antibiotic prescriptions were found in the low PMI group (*p* < 0.001).

### Laboratory findings of elderly COVID-19 patients according to PMI

In comparison to normal PMI patients, the levels of hemoglobin were lower in low PMI patients (122 g/L, IQR 117–129 vs. 129 g/L, IQR 122–137.5, *p* = 0.009). There was no significant difference in levels of other laboratory indexes (*p* > 0.05) (See additional file 1).

### CT findings of COVID-19 induced lung injury

As shown in Table 2, there were no significant differences of CTSS1, CTSS2 and POS on T1 between two group (*p* > 0.05). The maximum scores (T2) of CTSS1, CTSS2 and POS in low PMI patients were higher than normal PMI patients (all *p* < 0.001). In comparison to normal PMI group, low PMI group had significantly higher T3 levels of CTSS1, CTSS2 and POS (all *p* < 0.001).

**Table 2:** CT scores of COVID-19 induced lung injury

	T1			T2			T3		
	Low PMI (n = 39)	Normal PMI (n = 77)	*p* value	Low PMI (n = 39)	Normal PMI (n = 77)	*p* value	Low PMI (n = 39)	Normal PMI (n = 77)	*p* value
CTSS 1	3(2,5)	3(2,4)	0.093	7(6,10)	5(3,6)	< 0.001	5(4,7)	4(3,5)	< 0.001
CTSS 2	4(2,5)	3(2,5)	0.192	8(7,11)	5(4,7)	< 0.001	6(5,8)	4(3,6)	< 0.001
POS	1(0,1)	0(0,1)	0.079	2(1,2.5)	1(1,2)	< 0.001	1(1,2)	1(0,1)	< 0.001
*Changes at T2 from T1*									
ΔCTSS1 (T2)	–	–	–	4(2,6)	1(1,3)	< 0.001	–	–	–
ΔCTSS2 (T2)	–	–	–	5(2,7)	2(1,4)	< 0.001	–	–	–
ΔPOS (T2)	–	–	–	1(1,2)	1(0,1)	0.002	–	–	–
*Changes at T3 from T1*									
ΔCTSS1 (T3)	–	–	–	–	–	–	2(1,3)	1(0,2)	0.002
ΔCTSS2 (T3)	–	–	–	–	–	–	3(1,5)	1(0,2)	< 0.001
ΔPOS (T3)	–	–	–	–	–	–	1(0,1)	0(0,1)	0.002

Data are the median (interquartile range)

In the fields of CT score change at T2 from T1 (ΔT2), we found the levels of ΔCTSS1 (T2) (*p* < 0.001), ΔCTSS2 (T2) (*p* < 0.001), ΔPOS (T2) (*p* = 0.002) in low PMI patients were higher than those of normal PMI patients. These significant differences were also found in CT score change at T3 from T1 in ΔCTSS1 (T3) (*p* = 0.002), ΔCTSS2 (T3) (*p* < 0.001) and ΔPOS (T3) (*p* = 0.002) (See additional file 1).

There were no significant differences between low PMI and normal PMI patients in terms of other CT features, such as parenchymal infiltrate level, longitudinal distribution ratio, axial distribution ratio, number of involved lobes and pleural effusions (*p* > 0.05) (See additional file 1).

### Correlation analysis

In order to evaluate the risk factors of severe lung injury in elderly COVID-19 patients, we performed univariate and multivariate logistic regression analysis. We set three severity cut-offs, which were CTSS1 > 8, CTSS2 > 8 and POS = 3. After adjusting the confounders (age, sex and comorbidities), we found that smoking history (OR = 11.57, 95%CI 2.05–65.23, *p* = 0.006), comorbidity (OR = 10.92, 95%CI 1.12–106.79, *p* = 0.040), and presence of low PMI (OR = 8.53, 95%CI 2.14–34.02, *p* = 0.002) were predictors of severe lung injury with CTSS1 > 8 (Table 3). Comorbidity (OR = 5.88, 95%CI 1.10–31.37, *p* = 0.038) and presence of low PMI (OR = 8.64, 95%CI 2.79–25.69, *p* < 0.001) were predictors of severe lung injury with CTSS2 > 8 (Table 4). Moreover, presence of low PMI (OR = 4.03, 95%CI 1.25–12.95, *p* = 0.019) was the predictor of severe lung injury with POS = 3 (Table 5).

**Table 3:** Univariate and multivariate analysis of variables for lung injury (CTSS1 > 8 points, n = 19)

Variable	Univariate analysis		Multivariate analysis	
	OR (95% CI)	*p* value	OR (95% CI)	*p* value
Age (years)	0.93(0.84,1.03)	0.161	–	–
Age ≥ 80	1.03(0.31,3.44)	0.966	0.28(0.055,1.44)	0.128
Male gender	0.45(0.14,1.47)	0.186	0.071(0.01,0.50)	0.008
Smoking history	3.42(1.20,9.78)	0.022	11.57(2.05,65.23)	0.006
*BMI*				
Overweight	1.30(0.45,3.78)	0.633	–	–
Obese	1.50(0.35,6.52)	0.589	–	–
Comorbidity	11.1(1.42,86.65)	0.022	10.92(1.12,106.79)	0.040
Hypertension	2.07(0.75,5.69)	0.161	–	–
Diabetes	3.42(1.19,9.78)	0.022	1.22(0.31,4.90)	0.775
Coronary heart disease	1.29(0.14,12.24)	0.823	–	–
COPD/asthma	1.31(0.26,6.71)	0.747	–	–
Carcinoma history	2.09(0.50,8.71)	0.313	–	–
NLR	0.97(0.84,1.13)	0.698	–	–
PMI	0.029 (0.94,1.13)	0.539	1.21(1.04,1.41)	0.016
Low PMI	5.92(2.04,17.2)	0.001	8.53(2.14,34.02)	0.002
D-dimer	0.38(0.08,1.77)	0.216	–	–
Fully vaccinated	0.64(0.13,3.08)	0.580	–	–

**Table 4:** Univariate and multivariate analysis of variables for lung injury (CTSS2 > 8 points, n = 26)

Variable	Univariate analysis		Multivariate analysis	
	OR (95% CI)	*p* value	OR (95% CI)	*p* value
Age (years)	0.94 (0.86,1.03)	0.173		
Age ≥ 80	1.58(0.57,4.36)	0.376	0.62(0.17,2.23)	0.465
Male gender	0.49(0.18,1.35)	0.170	0.37(0.11,1.20)	0.096
Smoking history	2.45(0.93,6.47)	0.071		
*BMI*				
Overweight	0.75(0.29,1.94)	0.552		
Obese	0.75(0.19,3.05)	0.688		
Comorbidity	8.00(1.78,35.96)	0.007	5.88(1.10,31.37)	0.038
Hypertension	2.00(0.82,4.88)	0.128		
Diabetes	3.42(1.20,9.78)	0.022	3.31(1.02,10.73)	0.046
Coronary heart disease	0.86(0.092,8.05)	0.895		
COPD/asthma	0.85(0.17,4.29)	0.848		
Carcinoma history	1.34(0.33,5.45)	0.685		
NLR	0.96(0.81,1.14)	0.617		
PMI	1.00(0.92,1.09)	0.948		
Low PMI	7.39(2.82,19.41)	< 0.001	8.64(2.79,25.69)	< 0.001
D-dimer	0.45(0.13,1.53)	0.198		
Fully vaccinated	1.08(0.32,3.64)	0.905

**Table 5:** Univariate and multivariate analysis of variables for lung injury (POS = 3 points, n = 16)

Variable	Univariate analysis		Multivariate analysis	
	OR (95% CI)	*p* value	OR (95% CI)	*p* value
Age (years)	0.97(0.88,1.08)	0.590		
Age ≥ 80	0.87(0.23,3.33)	0.837	0.43(0.10,1.84)	0.254
Male gender	0.84(0.27,2.62)	0.770	0.73(0.22,2.48)	0.617
Smoking history	1.82(0.57,5.83)	0.315		
*BMI*				
Overweight	1.54(0.48,4.94)	0.472		
Obese	2.08(0.46,9.55)	0.345		
Comorbidity	8.81(1.12,69.43)	0.039	7.99(0.93,68.64)	0.058
Hypertension	1.96(0.66,5.80)	0.226		
Diabetes	5.08(1.93,13.39)	0.001	1.40(0.41,4.77)	0.590
Coronary heart disease	1.60(0.17,15.30)	0.683		
COPD/asthma	0.67(0.08,5.71)	0.718		
Carcinoma history	1.44(0.28,7.39)	0.659		
NLR	0.99(0.88,1.10)	0.796		
PMI	1.07(0.97,1.17)	0.186		
Low PMI	4.08(1.36,12.26)	0.012	4.03(1.25,12.95)	0.019
D-dimer	0.35(0.061,1.97)	0.232		
Fully vaccinated	0.81(0.17,3.93)	0.793		

Then, evaluated the predictors of the aggravation of lung injury by a univariate followed multivariate logistic regression method. We analyzed changes of CT score at T2 from T1 and set three severity cut-offs, including ΔCTSS1 > 4, ΔCTSS2 > 4 and ΔPOS = 2. After adjusting the confounders (age, sex and comorbidities), we found that comorbidity (OR = 6.15, 95%CI 1.28–29.54, *p* = 0.023) and the presence of low PMI (OR = 5.43, 95%CI 1.95–15.07, *p* = 0.001) were predictors of lung injury aggravation with ΔCTSS1 > 4. Presence of low PMI (OR = 5.98, 95%CI 2.35–15.22, *p* < 0.001) was the predictor of lung injury aggravation with ΔCTSS2 > 4. Meanwhile, the presence of low PMI (OR = 2.82, 95%CI 1.04–7.66, *p* = 0.042) and incrementally increased D-dimer level (OR = 0.088, 95%CI 0.011–0.729, *p* = 0.024) were predictors of lung injury aggravation with ΔPOS = 2 (See additional file 1).

## Discussion

In this retrospective single-center study, we evaluated 116 elderly patients from Nanjing Public Health Medical Center, the only designated hospital for COVID-19 in Nanjing. All the patients were linked to the outbreak of COVID-19 in Nanjing Lukou international airport in June 2021. The main finding was the association between severity of COVID-19 induced lung injury and lower-than- first-tertile PMI (16.4 and 13.8 cm^2^/m^2^ for males and females, respectively) measured on chest CT performed upon admission. Low PMI is significantly associated with pneumonia severity CT score and is an independent indication for severe lung injury. To the best of our knowledge, this is the first study to assess the role of PMI on chest CT for lung injury prognosis in elderly COVID-19 patients.

In COVID-19 patients, advanced age and various pre-existing comorbidities have been related with higher risk of adverse prognosis [22, 23]. With the increase of age and prevalence of chronic diseases, sarcopenia is generally found in elderly subjects [8]. Lower muscle mass or sarcopenia is negative predictors for severe COVID-19 [24]. Impaired respiratory muscle status has long been related to higher risk in pneumonia [25, 26]. Chest CT is a promising way to evaluate respiratory muscle mass. It has been reported that lower mass of paravertebral muscles on CT is independently associated with ICU admission and hospital mortality in COVID-19 patients [15]. Considering the bias related to the measurement of paravertebral muscle mass caused by gravity pressure in supine position, we measured area of pectoralis muscle on chest CT. Our results showed that Low PMI group was older and had more likely to be active smokers than normal PMI group. Low PMI group showed a longer day from T1 to T2 than normal group, which indicated that low PMI patients presented a prolonged peak in lung injury. In the fields of COVID-19 syndrome, we calculated the clinical syndrome score in each patient, which was the sum of each symptom duration days. We found that the clinical syndrome score was higher in low PMI group than normal group. Low PMI patients were more likely to present syndromes of nausea/vomiting, congested/runny nose and pharyngeal discomfort. All these results indicated that elderly low PMI patients suffered heavier COVID-19 clinical syndromes. Congested/runny nose and pharyngeal discomfort are common syndromes in upper respiratory tract infection. Many reasons can probably cause nausea and vomiting in COVID-19, including ACE2-mediated SARS-CoV-2 invasion of gastrointestinal epithelium and systemic inflammatory response [27]. It needs a further investigation on whether and how impaired muscle status enhance afferent pathways arise from cerebral cortex to trigger nausea and vomiting.

Our laboratory findings indicated that the levels of hemoglobin were lower in low PMI patients. Decrease of hemoglobin in the elderly is attributable to nutritional deficiency, including vitamin B12, folate, and iron deficiency. Sarcopenia or low muscle mass patients are more likely to have lower levels of hemoglobin [28]. Furthermore, recent studies suggested that 19–38% patients with COVID-19 presented decreased hemoglobin concentration [29, 30]. A meta-analysis enrolled 189 studies and 57,563 COVID-19 patients indicated a pooled mean hemoglobin concentration of 129.7 g/L, which decreased with older age and a higher proportion of comorbid illness and disease severity [31]. Thus, level of hemoglobin deserves attention in elderly COVID-19 patients with impaired muscle status.

In term of lung injury evaluation, we performed a dynamic chest CT observation and choose three CT severity score methods. All the enrolled elderly COVID-19 patients underwent at least 5 times chest CT test in 2 weeks or so after admission. Our results showed that low PMI patients had higher maximum scores (T2) of CTSS1, CTSS2 and POS, which mean a more severe lung injury. These significantly higher scores could be still observed at 2 weeks or so after admission (T3) in low PAI patients. CTSS is based on a subjective visual assessment of the extent of injury, including GGO, crazy paving, or consolidation, in each part of the lung. There are five lobes in CTSS 1 and six zones in CTSS 2, therefore their total scores range from 0 to 20 and 0 to 24, respectively. Independent of conventional risk factors of COVID-19, a study of 210 confirmed COVID-19 patients indicated that extensive lung damage could be visually assessed with CTSS 1 related to 30-day mortality [32]. Another retrospective cohort study enrolled 262 hospitalized COVID-19 patients showed that mortality was significantly higher in patients with higher CTSS2 even after adjustment for clinical, demographics and laboratory parameters [21]. POS was calculated as the percentage of pulmonary opacity area relative to the entire lung on CT images. POS upon admission was closely related to some clinical characteristics and was an independent predictor of disease severity, ICU admission, respiratory failure and long hospital stay in patients with COVID-19 [19]. There are few studies focus on elderly COVID-19 patients in the relationship with lung injury CT score and impaired muscle status. Our results indicated that low PMI is an independent risk factor for severe lung injury under all of three CT scores after adjusting age and male. Hocaoglu et al. reported that CT-derived measurements of the pectoralis muscle could be useful in predicting disease severity and mortality rate of COVID-19 pneumonia in adult patients [33]. In Hocaoglu's study, they analyzed the pectoralis muscle volume and pectoralis muscle density of adult COVID-19 patients. But the data did not corrected to BSA or others. Ufuk et al. found PMI value was a predictor of prolonged hospital stay and death for COVID-19 patients [17]. In Ufuk’s study, they did not specifically analyze elderly COVID-19 patients and the value of PMI was corrected to height square but not BSA. We believed that a correction of BSA would be more appropriate. Our results did not show PMI had any association to death or prolonged hospital stay, which might be due to the fact that most of our patients were common type of COVID-19.

Some limitations of our study should be considered, other than its retrospective nature. First, there are few severe COVID-19 patients in this 2020 Nanjing COVID-19 epidemic. Only 3 patients in our study were severe cases. It still needs further studies to confirm the value of CT-derived PMI in the predicting disease severity and mortality rate of COVID-19 pneumonia in adult patients. Second, as a retrospective study, we were not able to perform muscle strength assessments, such as handgrip strength test, and acquired the information of daily physical activity in each elderly COVID-19 patient. Third, we only analyzed pectoralis muscle aera on chest CT. There is a lack of other supporting evidence of muscle mass, such as vastus lateralis which assessed by ultrasound. Fourth, in a short period of time, there were many patients sent to Nanjing Public Health Medical Center, the only designated hospital provides medical services for COVID-19 in Nanjing. Owing to limited medical resources, we were not able to routinely test testosterone level of each patient. As testosterone level in elderly patients decreases and varies from person to person, and testosterone level impacts muscle mass, which is the confounder in our study. Larger scale studies are needed to further confirm the association of muscle status and COVID-19.

## Conclusions and implications

In conclusion, our results suggest that PMI measured in chest CT is associated to the severity of lung injury in elderly COVID-19 patients. PMI can be easily assessed on chest CT images of COVID-19 patients.

## Supplementary Information

**Additional file 1. Table S1.** Laboratory findings of elderly COVID-19 patients according to PMI. **Table S2.** Univariate and multivariate analysis of variables for change of CT score from T1 to T2 (ΔCTSS1 > 4 points, n = 24). **Table S3.** Univariate and multivariate analysis of variables for change of CT score from T1 to T2 (ΔCTSS2 > 4 points, n = 34). **Table S4.** Univariate and multivariate analysis of variables for change of CT score from T1 to T2 (ΔPOS = 2 points, n = 25). **Table S5.** ICC values (95% CI). **Table S6.** CT findings of COVID-19 induced lung injury.

## Data Availability

The datasets used and/or analyzed during the current study available from the corresponding author on reasonable request.

## Abbreviations

SARS-CoV-2: Severe acute respiratory syndrome coronavirus 2
COVID-19: Coronavirus disease 2019
PMI: Pectoralis muscle index
PMA: Pectoralis muscle area
CTSS: CT severity score
GGO: Ground-glass opacities
