# Supplementary material for: Correlation of CT-derived pectoralis muscle status and COVID-19 induced lung injury in elderly patients
Source: BMC Med Imaging. 2022 Aug 12;22:144. doi: 10.1186/s12880-022-00872-9 (PMC9372984; doi:10.1186/s12880-022-00872-9)
Supplement: Supplementary file 1 — Additional file 1. Table S1. Laboratory findings of elderly COVID-19 patients according to PMI. Table S2. Univariate and multivariate analysis of variables for change of CT score from T1 to T2 (ΔCTSS1 > 4 points, n = 24). Table S3. Univariate and multivariate analysis of variables for change of CT score from T1 to T2 (ΔCTSS2 > 4 points, n = 34). Table S4. Univariate and multivariate analysis of variables for change of CT score from T1 to T2 (ΔPOS = 2 points, n = 25). Table S5. ICC values (95% CI). Table S6. CT findings of COVID-19 induced lung injury. [file 12880_2022_872_MOESM1_ESM.docx]

Table S1 Laboratory findings of elderly COVID-19 patients according to PMI

| characteristics | All Patients  (N=116) | Low PMI  (n=39) | Normal PMI(n=77) | p value |
| --- | --- | --- | --- | --- |
| Inflammatory parameters |  |  |  |  |
| PCT | 0.05(0.03,0.08) | 0.06(0.03,0.08) | 0.05(0.04,0.08) | 0.817 |
| HS-CRP | 8.6(3.3,23.7) | 11.4(5.2, 23.7) | 6.8(3.0, 24.7) | 0.108 |
| IL-6 | 22.2(13.6,35.2) | 24.6(16.8,34.3) | 21.5(12.0,37.1) | 0.300 |
| Blood cells |  |  |  |  |
| WBCs × 10^9^/L | 4.3(3.6,5.4) | 4.6(3.9,5.5) | 4.1(3.5,5.4) | 0.308 |
| Neutrophils /% | 61.4(10.4) | 62.5(9.8) | 63.7(10.8) | 0.225 |
| Lymphocytes /% | 25.5(9.5) | 26.4(8.9) | 25.0(9.8) | 0.236 |
| NLR/% | 2.4(1.7,3.8) | 2.3(1.8,3.4) | 2.6(1.7,4.1) | 0.577 |
| Platelets × 10^9^/L | 127.5(98,163) | 138(114,165) | 121(96.5,162) | 0.217 |
| Haemoglobin, g/L | 126(120,135) | 122(117,129) | 129(122,137.5) | 0.009 |
| Coagulation parameters |  |  |  |  |
| Prothrombin time, s | 11.1(10.8,11.7) | 11.2(10.7,11.6) | 11.1(10.8,11.7) | 0.872 |
| Activated partial thromboplastin time, s | 27.1(24.7,29.9) | 27.0(25,29.7) | 27.4(24.3,30.1) | 0.953 |
| D-dimer, μg/ml | 0.46(0.28,0.72) | 0.41(0.31,0.69) | 0.48(0.28,0.78) | 0.537 |
| Blood biochemistry |  |  |  |  |
| Albumin, g/L | 39.8(37.4,41.5) | 39.2(37.3, 41.0) | 39.8(37.7, 42.5) | 0.092 |
| Alanine aminotransferase, U/L | 23.0(17.9, 32.5) | 22.2(16.3, 32.0) | 23.0(19.2, 34.7) | 0.212 |
| Aspartate aminotransferase, U/L | 22.7(16.2, 32.4) | 22.2(16.3, 32.0) | 23.0(19.2, 34.7) | 0.237 |
| Total bilirubin, mmol/L | 10.2(7.7, 13.2) | 10.6(7.1, 13.8) | 10.1(8.1, 13.0) | 0.752 |
| Blood urea nitrogen, mmol/L | 5.1(4.2, 6.1) | 5.1(3.9, 6.2) | 5.1(4.3, 5.9) | 0.739 |
| Serum creatinine, μmol/L | 65.8(56.0, 78.3) | 67.0(58.4, 78.1) | 65.4(55.8, 78.6) | 0.779 |
| Lactate dehydrogenase, U/L | 252(224.3, 306.3) | 246.0(225.0, 321.0) | 256.0(224.0, 298.5) | 0.695 |
| Troponin, pg/mL | 10.0 (6.1, 15.3) | 10.0(8.4, 15.3) | 10.0(5.6, 15.4) | 0.610 |
| Glucose, mmol/L | 5.8(4.8, 7.3) | 5.6(4.7, 7.3) | 5.8(4.9, 7.3) | 0.518 |

Note—Data are the median (interquartile range) or number of patients (percentage) unless otherwise indicated. NLR= neutrophil to lymphocyte ratio

Table S2 Univariate and multivariate analysis of variables for change of CT score from T1 to T2 (ΔCTSS1 > 4 points, n=24)

|  | Univariate Analysis | | Multivariate Analysis | |
| --- | --- | --- | --- | --- |
| Variable | OR (95% CI) | p-value | OR (95% CI) | p-value |
| Age (years) | 0.916(0.833,1.01) | 0.071 |  |  |
| Age ≥ 80 | 2.38(0.87,6.49) | 0.092 | 1.30(0.42,4.04) | 0.651 |
| Male gender | 0.74(0.28,1.96) | 0.539 | 0.63(0.21,1.88) | 0.410 |
| Smoking history | 1.28(0.45,3.67) | 0.645 |  |  |
| BMI |  |  |  |  |
| Overweight | 1.32(0.51,3.41) | 0.562 |  |  |
| Obese | 0.63(0.12,3.21) | 0.577 |  |  |
| Comorbidity | 7.07(1.57,31.91) | 0.011 | 6.15(1.28,29.54) | 0.023 |
| Hypertension | 2.07(0.82,5.22) | 0.122 |  |  |
| Diabetes | 2.56(0.83,7.91) | 0.103 |  |  |
| Coronary heart disease | 0.96(0.10,8.98) | 0.969 |  |  |
| COPD/asthma | 0.40(0.048,3.33) | 0.397 |  |  |
| Carcinoma history | 2.43(0.65,9.11) | 0.188 |  |  |
| NLR | 0.93(0.74,1.18) | 0.556 |  |  |
| PMI | 0.98(0.90,1.07) | 0.633 |  |  |
| Low PMI | 6.0(2.27,15.85) | <0.001 | 5.43(1.95,15.07) | 0.001 |
| D-dimer | 0.43(0.12,1.58) | 0.202 |  |  |
| Fully vaccinated | 0.47(0.099,2.20) | 0.335 |  |  |

Table S3 Univariate and multivariate analysis of variables for change of CT score from T1 to T2 (ΔCTSS2 > 4 points, n=34)

|  | Univariate Analysis | | Multivariate Analysis | |
| --- | --- | --- | --- | --- |
| Variable | OR (95% CI) | p-value | OR (95% CI) | p-value |
| Age (years) | 0.95(0.88,1.03) | 0.204 |  |  |
| Age ≥ 80 | 1.27(0.49,3.32) | 0.627 | 0.63(0.20,1.96) | 0.419 |
| Male gender | 0.59(0.25,1.43) | 0.245 | 0.49(0.18,1.31) | 0.155 |
| Smoking history | 1.18(0.45,3.06) | 0.739 |  |  |
| BMI |  |  |  |  |
| Overweight | 1.50(0.63,3.57) | 0.359 |  |  |
| Obese | 1.50(0.44,5.14) | 0.519 |  |  |
| Comorbidity | 2.99(1.11,8.01) | 0.030 | 2.32(0.74,7.30) | 0.149 |
| Hypertension | 1.54(0.69,3.45) | 0.293 |  |  |
| Diabetes | 3.67(1.37,9.79) | 0.010 | 2.03(0.69,6.01) | 0.201 |
| Coronary heart disease | 0.59(0.064,5.49) | 0.644 |  |  |
| COPD/asthma | 0.58(0.12,2.88) | 0.503 |  |  |
| Carcinoma history | 1.43(0.39,5.24) | 0.591 |  |  |
| NLR | 0.84(0.66,1.07) | 0.156 |  |  |
| PMI | 0.97(0.90,1.05) | 0.472 |  |  |
| Low PMI | 5.74(2.41,13.67) | <0.001 | 5.98(2.35,15.22) | <0.001 |
| D-dimer | 0.96(0.57,1.61) | 0.878 |  |  |
| Fully vaccinated | 0.71(0.21,2.35) | 0.572 |  |  |

Table S4 Univariate and multivariate analysis of variables for change of CT score from T1 to T2 (ΔPOS = 2 points, n=25)

|  | Univariate Analysis | | Multivariate Analysis | |
| --- | --- | --- | --- | --- |
| Variable | OR (95% CI) | p-value | OR (95% CI) | p-value |
| Age (years) | 0.93(0.85,1.02) | 0.119 |  |  |
| Age ≥ 80 | 0.68(0.21,2.20) | 0.515 | 0.46(0.13,1.67) | 0.237 |
| Male gender | 0.87(0.34,2.23) | 0.768 | 0.82(0.29,2.29) | 0.702 |
| Smoking history | 1.20(0.42,3.41) | 0.737 |  |  |
| BMI |  |  |  |  |
| Overweight | 2.12(0.81,5.55) | 0.125 |  |  |
| Obese | 1.31(0.31,5.58) | 0.719 |  |  |
| Comorbidity | 3.13(0.99,9.90) | 0.052 | 3.27(0.97,11.05) | 0.057 |
| Hypertension | 1.49(0.61,3.62) | 0.384 |  |  |
| Diabetes | 2.05(0.76,5.52) | 0.157 |  |  |
| Coronary heart disease | 0.91(0.097,8.49) | 0.931 |  |  |
| COPD/asthma | 0.38(0.046,3.15) | 0.370 |  |  |
| Carcinoma history | 1.42(0.35,5.78) | 0.629 |  |  |
| NLR | 0.92(0.73,1.18) | 0.516 |  |  |
| PMI | 1.01(0.92,1.10) | 0.895 |  |  |
| Low PMI | 2.71(1.09,6.71) | 0.031 | 2.82(1.04,7.66) | 0.042 |
| D-dimer | 0.11(0.017,0.73) | 0.022 | 0.088(0.011,0.729) | 0.024 |
| Fully vaccinated | 0.75(0.20,2.85) | 0.673 |  |  |

Table S5 ICC values (95% CI)

|  | T0 | T1 | T2 |
| --- | --- | --- | --- |
| CTSS1 | 0.991 (0.986-0.993) | 0.995 (0.992-0.996) | 0.994 (0.991-0.996) |
| CTSS2 | 0.988 (0.983-0.992) | 0.996 (0.994-0.997) | 0.992 (0.988-0.994) |
| POS | 0.954(0.934-0.968) | 0.952 (0.932-0.967) | 0.958 (0.941-0.971) |

Table S6 CT findings of COVID-19 induced lung injury

|  | T1 |  |  | T2 |  |  | T3 |  |  |
| --- | --- | --- | --- | --- | --- | --- | --- | --- | --- |
|  | Low PMI  (n=39) | Normal PMI(n=77) | P value | Low PMI  (n=39) | Normal PMI(n=77) | P value | Low PMI  (n=39) | Normal PMI(n=77) | P value |
| Parenchymal infiltrate |  |  | 0.351 |  |  | 0.257 |  |  |  |
| Normal | 4(7.7) | 6(7.8) |  | 0 (0) | 1(1.3) |  | 0(0) | 1(1.2) | 0.563 |
| GGO | 23(59.0) | 42(54.5) |  | 7(17.9) | 24(31.2) |  | 13(33.3) | 33(42.9) |  |
| Consolidation | 8(20.5) | 25(32.5) |  | 22(56.4) | 40(51.9) |  | 11(28.2) | 20(26.0) |  |
| Both | 5(12.8) | 4(5.2) |  | 10(25.7) | 12(15.6) |  | 15(38.5) | 23(29.9) |  |
| Longitudinal distribution |  |  | 0.171 |  |  | 0.508 |  |  | 0.390 |
| none | 3(7.7) | 5(6.5) |  | 0(0) | 0(0) |  | 0(0) | 1(1.3) |  |
| Upper zone | 7(17.9) | 5(6.5) |  | 1(2.6) | 3(3.9) |  | 2(5.1) | 3(3.9) |  |
| Middle zone | 1(2.7) | 0(0) |  | 1(2.6) | 0(0) |  | 2(5.1) | 1(1.3) |  |
| Lower zone | 7(17.9) | 13(16.9) |  | 3(7.6) | 6(7.8) |  | 3(7.7) | 13(16.9) |  |
| Random | 21(53.8) | 54(70.1) |  | 34(87.2) | 68(88.3) |  | 32(82.1) | 59(76.6) |  |
| Axial distribution |  |  | 0.961 |  |  | 0.112 |  |  | 0.221 |
| none | 3(7.7) | 5(6.5) |  | 0(0) | 0(0) |  | 0 | 1(1.3) |  |
| Peripheral | 25(64.1) | 51(66.2) |  | 17(43.6) | 47 (61.0) |  | 19(48.7) | 48(62.3) |  |
| Central | 0 | 0 |  | 0 | 0 |  | 0 | 0 |  |
| Diffuse | 11(28.2) | 21(27.3) |  | 22(56.4) | 30(39.0) |  | 20(51.3) | 28(36.4) |  |
| No. of lobes |  |  | 0.841 |  |  | 0.614 |  |  | 0.779 |
| 0 | 3(7.7) | 4(5.2) |  | 0(0) | 0(0) |  | 0(0) | 0(0) |  |
| 1 | 8(20.5) | 11(14.3) |  | 1(2.6) | 1(1.3) |  | 2(5.1) | 6(7.8) |  |
| 2 | 7(17.9) | 17(22.1) |  | 3(7.7) | 8(10.4) |  | 4(10.3) | 12(15.5) |  |
| 3 | 10(25.7) | 18(23.4) |  | 6(15.4) | 18(23.4) |  | 9(23.1) | 16(20.8) |  |
| 4 | 8(20.5) | 16(20.8) |  | 7(17.9) | 17(22.1) |  | 10(25.6) | 13(16.9) |  |
| 5 | 3(7.7) | 11(14.2) |  | 22(56.4) | 33(42.9) |  | 14(35.9) | 30(39.0) |  |
| Pleural effusions | 3(7.7) | 2(2.6) | 0.333 | 5(12.8) | 5(6.5) | 0.300 | 5(12.8) | 6(7.8) | 0.504 |

Note: Data are number of patients (percentage).
